# The expression dynamics of IL-17 and Th17 response relative cytokines in the trachea and spleen of chickens after infection with *Cryptosporidium baileyi*

**DOI:** 10.1186/1756-3305-7-212

**Published:** 2014-05-06

**Authors:** Guang-Hui Zhao, Wen-Yu Cheng, Wan Wang, Yan-Qing Jia, Yan-Qin Fang, Shuai-Zhi Du, San-Ke Yu

**Affiliations:** 1 College of Veterinary Medicine, Northwest A&F University, Yangling, Shaanxi Province 712100, People’s Republic of China; 2 College of Life Sciences, Northwest A&F University, Yangling, Shaanxi Province 712100, People’s Republic of China

**Keywords:** *Cryptosporidium baileyi*, IL-17, Chicken, Immunity

## Abstract

**Background:**

*Cryptosporidium baileyi* is the dominant *Cryptosporidium* species in birds causing emerging health problems in the poultry industry, and is also a model to study the biology of *Cryptosporidium* spp.. IL-17 (also called IL-17A) is a hallmark pro-inflammatory cytokine of Th17 cells that plays an important role in several human autoimmune diseases and microbial infection disease of many animals, and it may play a role in *Cryptosporidium* infection.

**Methods:**

The present study examined the mRNA level of IL-17 and Th17 response relative cytokines in the trachea and spleen of *C. baileyi*-infected chickens at different time points using real-time quantitative PCR (qPCR).

**Results:**

All examined cytokines in the trachea were up-regulated in the infected chickens compared with the uninfected control during *C. baileyi* infection. Significant increased IL-17 mRNA level in the trachea was observed as early as 12 h post infection (pi), peaking at 24 h pi and 10 d pi, and declining thereafter. The transcription levels of IL-17 and Th17 response relative cytokines in spleen were also significantly increased at different time points during the infection.

**Conclusions:**

IL-17 was indicated to participate in the induction of inflammation during infection of some intracellular protozoan parasites. The results in the present study suggest that IL-17 may play a role in immunity against *Cryptosporidium* infection, and provide basic information for determining the role of Th17 cell in *Cryptosporidium* infection.

## Background

*Cryptosporidium* spp. are monoxenous protozoan parasites that inhabit microvilli of epithelial cells in digestive, respiratory and urinary tracts of a wide range of vertebrates [1-3], causing acute, self-limiting gastroenteritis in immunocompetent individuals, and chronic or potentially fatal infections in immunocompromised subjects worldwide [4-6]. More than 240 vertebrate species have been identified with *Cryptosporidium* infection, including livestock and human. However, no effective chemotherapy or preventive interventions are available for the control of cryptosporidiosis [7]. Considering that the infection status and severity of cryptosporidiosis are closely related to immunity of individuals, the knowledge of the host immune mechanisms against *Cryptosporidium* infection is essential for controlling this disease.

Previous studies have suggested that host resistance against *C. parvum* infection is established through both innate and adaptive immune responses [8,9]. Intestinal epithelial cells (IECs), natural killer (NK) cells, phagocytes, dendritic cells (DCs), interferon-γ, nitric oxide and the complement system play some protective roles at the beginning of infection [9]. Evidence from studies on *C. parvum* infections of murine, bovine, and humans suggested an important role for T cell-derived cytokines in the recovery from *Cryptosporidium* infection [9-12]. The importance of CD4^+^ T cells in immunity to *Cryptosporidium* infection has been demonstrated in murine infection models of *C. parvum *[9,13,14]. Subsequent findings demonstrated that both Th1 and Th2 cells have roles in controlling *C. parvum* infection, and there is a strong Th1 response during early infection but the later maturation of a more balanced response with a Th2 component may facilitate parasite removal [7,14]. However, an infection study using a knock out mouse model revealed that hosts lacking IFN-γ and IL-4 could still finally eliminate the parasites [15].

Recently, a new T-cell subset, largely produced by activated CD4^+^T cells distinct from Th1 or Th2 cells, designated as Th17, was identified [16,17]. A hallmark of this Th-cell subset is the production of IL-17A (also called IL-17), a pro-inflammatory cytokine that plays an important role in several diseases as well as Th17 cells [18]. Factors that promote Th17 cell differentiation and/or expansion are transforming growth factor (TGF)-β, IL-6 and IL-23 [17,19]. The differentiation is regulated by the transcription factor signal transducer and activator of transcription 3 (STAT-3), retinoic acid receptor-related orphan receptor-γτ (RORγτ) and aryl hydrocarbon receptor [20]. IL-17 promotes inflammation by enhancing production of cytokines such as IL-1β, TNF-α, IL-6 and receptor activation of nuclear factor-κ B ligand (RANKL) [21,22]. Th17 cells are thought to increase inflammation by recruiting cells, particularly neutrophils, to the peripheral tissues for pathogen clearance [23].

Although IL-17 has a role in the host’s protection against fungal and bacterial infection, the roles of IL-17 in host defense against intracellular protozoan parasites remain to be fully elucidated [24-26]. Several infection studies with various protozoal species demonstrated that Th17 cells may mediate the host defense against *Trypanosoma cruzi *[27], *Pneumocystis carinii *[28] and *Toxoplasma gondii *[29]. However, controversial results have been shown in human cutaneous leishmaniasis [30], *T. gondii* infection [31] and *Eimeria tenella* infection in chicken [32], which suggests that IL-17 contributes to the pathology of these infections.

*Cryptosporidium baileyi*, the dominant species and more frequently associated with high morbidity and mortality [33,34] in birds, mainly parasitizes the respiratory tract (larynx, trachea, primary and secondary bronchi and air sacs), bursa of Fabricius and cloaca of chickens, turkeys and ducks, and causes respiratory disorders, clinically characterized by rales, coughing, dyspnoea, and sneezing [33,35,36]. Ditrich *et al*. [37] also detected *C. baileyi* in the stool of an immunodeficient man, suggesting the zoonotic fact of this species. Considering the morphological and biological features and large oocyst production, *C. baileyi* has been suggested as a model for characterization of cryptosporidia [38]. Since *C. baileyi* represents a kind of respiratory intracellular parasite and can be an ideal infection model for study of respiratory infection, the expression dynamics of IL-17 and Th17 response relative cytokines in the trachea and spleen of chickens after infection with *C. baileyi* were studied. The results will have important implications for determining the role of IL-17 and Th17 cell in *Cryptosporidium* infection.

## Methods

### Ethics statement

The performance of this study was strictly according to the recommendations of the Guide for the Care and Use of Laboratory Animals of the Ministry of Health, China, and our protocol was reviewed and approved by the Research Ethics Committee of Northwest A&F University.

### Chickens

One-day-old Arbor Acre (AA) broiler cockerels purchased from the Youming Broiler Hatchery (Shaanxi, China) were reared in a coccidia-free laboratory, housed in wire cages, and given free access to feed and water and constant light during the entire experimental period. Chickens were randomly assigned to four groups of 10 birds per group (5 birds per cage). All animal experiments were carried out in compliance with current Chinese ethical legislation.

### Parasites

The wild-type strain of *C. baileyi* was originally isolated from a natural infected chick and identified using microscopic and molecular analyses. Oocysts were maintained by passage every two months in susceptible chickens at the College of Veterinary Medicine, Northwest A&F University, China.

### Parasite infection and measurements of fecal oocyst shedding

A total of 20 4-day-old chickens were each inoculated orally with a single dose of 1 × 10^5^*C. baileyi* oocysts (in 100 μL of PBS). Meanwhile, a total of 20 uninfected age-matched chickens served as control group and were given 100 μL PBS. For the determination of fecal oocyst shedding, birds (10 per group) were placed in oocyst collection cages and fecal samples were collected daily following inoculation. Numbers of oocyst per gram (OPG) of feces per group sample were determined using a hemacytometer according to the following formula: OPG/group = [(oocyst count/4) × 10^4^ × 5 × 5]/weight of fecal sample.

### RNA extraction and cDNA synthesis

Five chickens from control and infected groups were euthanized by cervical dislocation at 3 h, 6 h, 12 h, 24 h, 48 h, 96 h, 7 d, 10 d, 13 d and 16 d post infection (pi), then spleen and trachea were isolated. Total RNA was isolated from <100 mg of the tissues previously snap-frozen in liquid nitrogen. The RNA samples were extracted using TRIzol agent (Invitrogen) following the manufacturer’s instructions, with the RNA purity and concentration determined by spectrophotometric absorbance at 260 and 280 nm. The RNA sample (0.5 μg) was reversely transcribed into cDNA using PrimeScript™ RT reagent Kit (TaKaRa, DALIAN).

### Quantitative real-time RT-PCR

Quantitative RT-PCR oligonucleotide primers for chicken cytokines and β-actin control are described by Zhang *et al*. [32]. qPCR reactions of the genes were performed on an iQ5 Real-Time PCR Detection System (Bio-Rad, USA) using UltraSYBR Mixture (CWBIO, China). Amplification and detection of specific products were performed with the following conditions: one cycle of 95°C for 3 min, followed by 40 cycles of 95°C for 15 s, 56°C for 30 s. Relative gene expression data were analyzed using the 2^-ΔΔCt^ method [39].

### Statistical analysis

The data was analyzed using SPSS software (SPSS 18.0 for Windows; SPSS, Chicago, IL, USA), with one-way analysis of variance followed by the Duncan’s multiple range test. All data were expressed as the mean ± SD of 5 chickens per group with three replicates per sample. Differences between groups were considered statistically significant at P < 0.05.

## Results and discussion

To determine the law of oocyst shedding by infected chickens, fecal samples were collected and examined daily post infection (pi) by oocyst counting according to the method described by Gao *et al*. [40]. Oocyst shedding in the pooled fecal samples was firstly observed on day 4 pi and the highest levels of oocyst shedding occurred on day 10 pi, 12 pi and 14 pi, peaking at day 10 pi and declining thereafter (Additional file 1). Clinical signs of respiratory disease, consisting of rales, sneezing, and dyspnea were observed in all inoculated birds from day 10 pi to 21 pi. Two deaths occurred in the infected group at day 10 pi and 12 pi, respectively.

**Figure 1:**
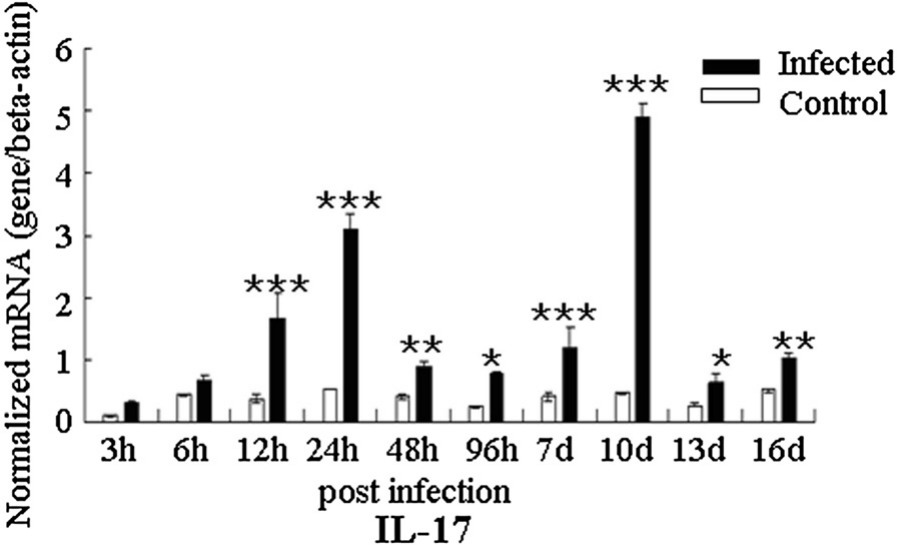
**Expression of IL-17 in trachea in *C. baileyi *infected chickens.** Chickens were orally infected with 1 × 10^5^ oocysts of *C. baileyi*. Tracheas were isolated from infected and non-infected chickens at the indicated times post-infection and mRNA levels were determined by qPCR (normalized to beta-actin transcript levels). Each bar represents the mean ± SD values (n = 5). ^*^means *P* < 0.05, ^**^means *P* < 0.01, ^***^means *P* < 0.001.

**Figure 2:**
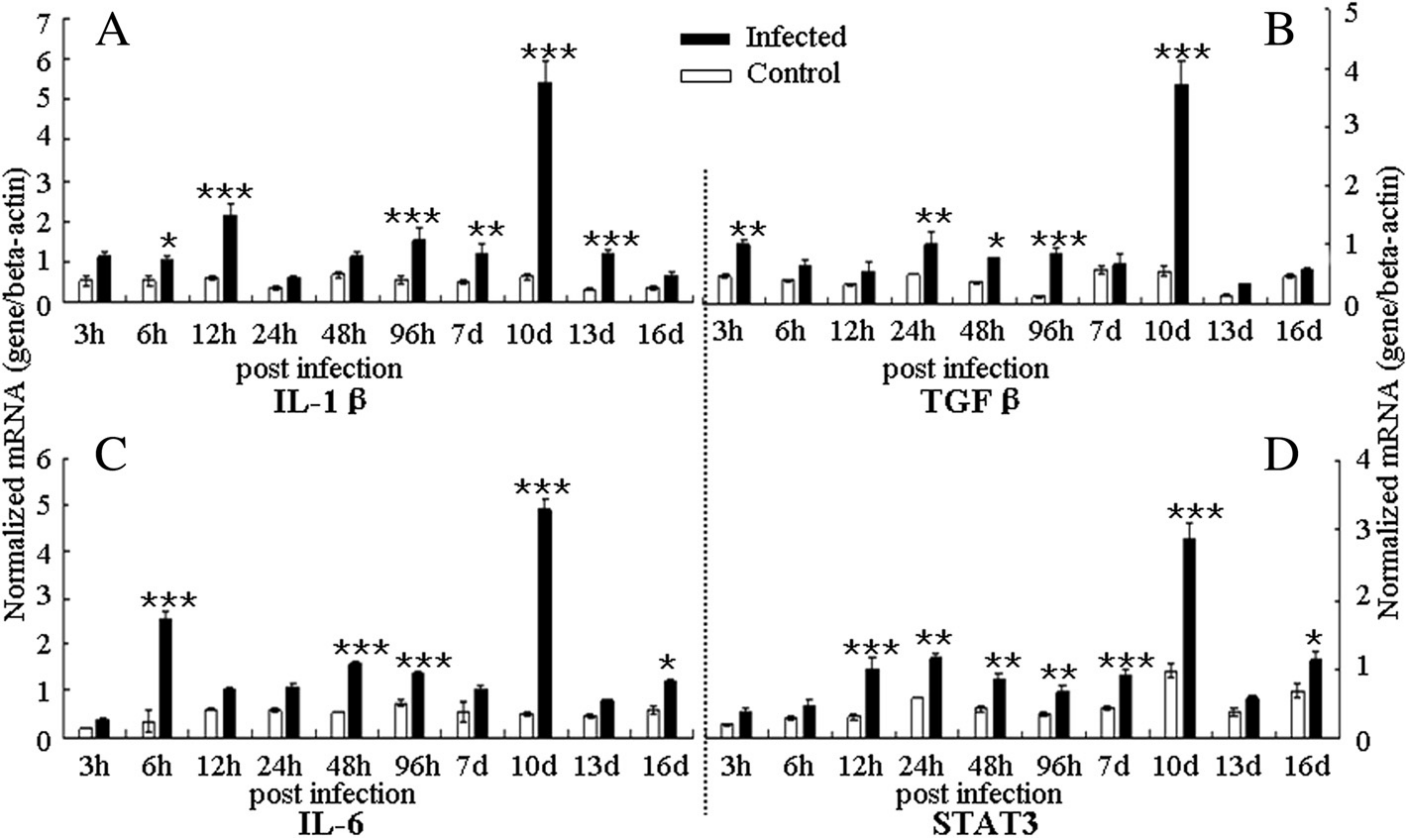
**Expression of Th17 response relative cytokines in** the **trachea in *C. baileyi *infected chickens.** Chickens were orally infected with 1 × 10^5^ oocysts of *C. baileyi*. Tracheas were isolated from infected and non-infected chickens at the indicated times post-infection and mRNA levels were determined by qPCR (normalized to beta-actin transcript levels). **(A)** IL-1β; **(B)** TGF-β; **(C)** IL-6; **(D)** STAT3; Each bar represents the mean ± SD values (n = 5). ^*^means *P* < 0.05, ^**^means *P* < 0.01, ^***^means *P* < 0.001.

The expression of IL-17 (Figure 1) and Th17 response relative cytokines (IL-1β, IL-6 and TGF-β, Figure 2) and transcription factor (STAT3) were detected in the trachea during the infection of *C. baileyi*. All these cytokines in the trachea were up-regulated in the infected chickens compared with the uninfected control during the *C. baileyi* infection. Significant increase in IL-17 mRNA expression was observed as early as 12 h pi, peaking at 24 h pi and 10 d pi, and declining thereafter. The expression level of STAT3, one of the key transcription factors for the polarization of Th17 cells, was also robustly up-regulated as early as 12 h pi, peaking at 10 d pi. For the expression of IL-6 and TGF-β, the early peaks were observed at 12 h pi and 6 h pi, respectively, while both of two cytokines have a peak at 10 d pi later. Interestingly, we observed significantly higher mRNA levels of all cytokines (IL-1β, IL-6, IL-17, TGF-β and STAT3) at 10 d pi, which is consistent with the oocyst shedding pattern (Additional file 1) in which the peak level of oocyst shedding was observed on day 10 pi.

**Figure 3:**
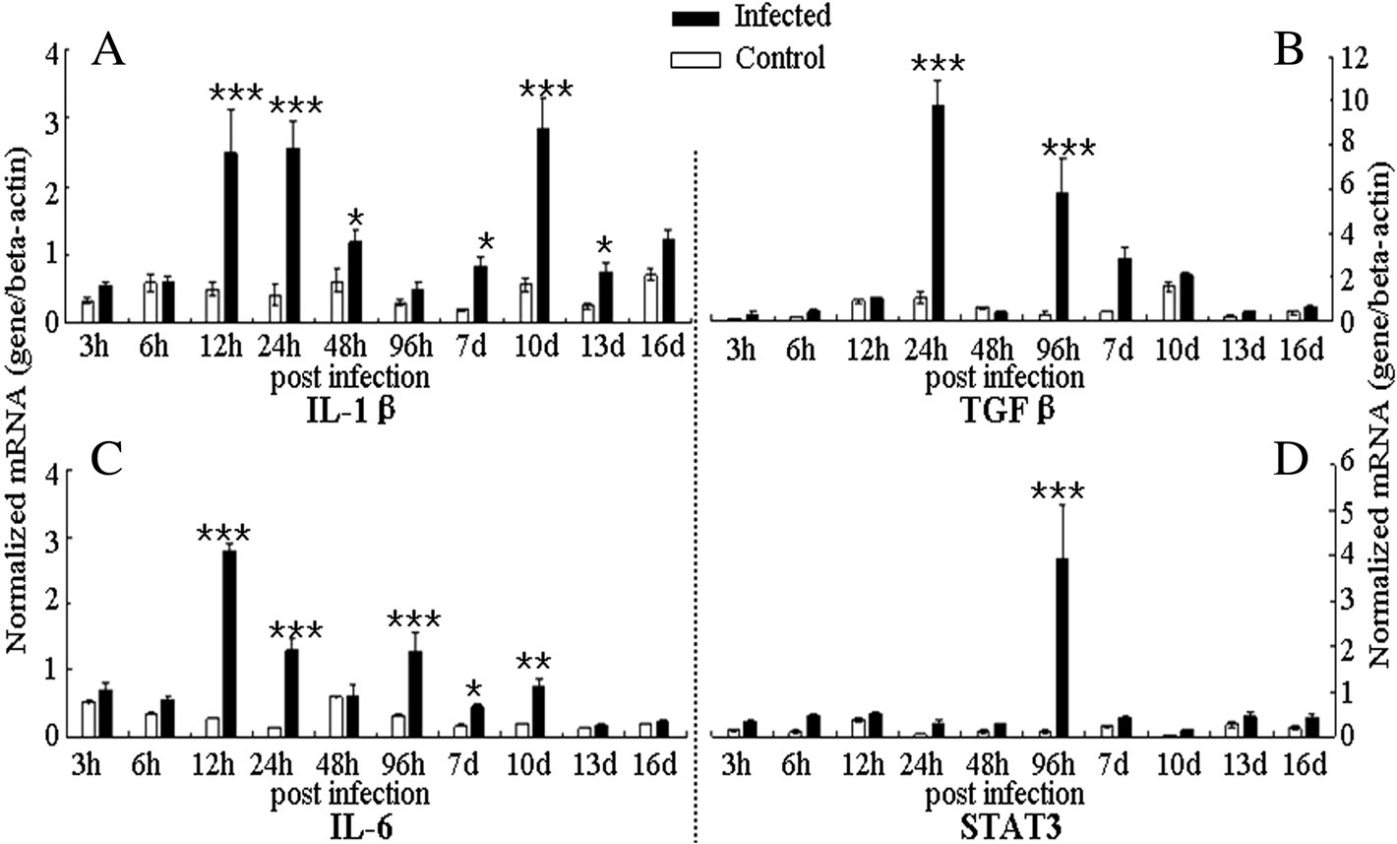
**Expression of Th17 response relative cytokines in spleen in *C. baileyi *infected chickens.** Chickens were orally infected with 1 × 10^5^ oocysts of *C. baileyi*. Spleens were isolated from infected and non-infected chickens at the indicated times post-infection and mRNA levels were determined by qPCR (normalized to beta-actin transcript levels). **(A)** IL-1β; **(B)** TGF-β; **(C)** IL-6; **(D)** STAT3; Each bar represents the mean ± SD values (n = 5). ^*^means *P* < 0.05, ^**^means *P* < 0.01, ^***^means *P* < 0.001.

**Figure 4:**
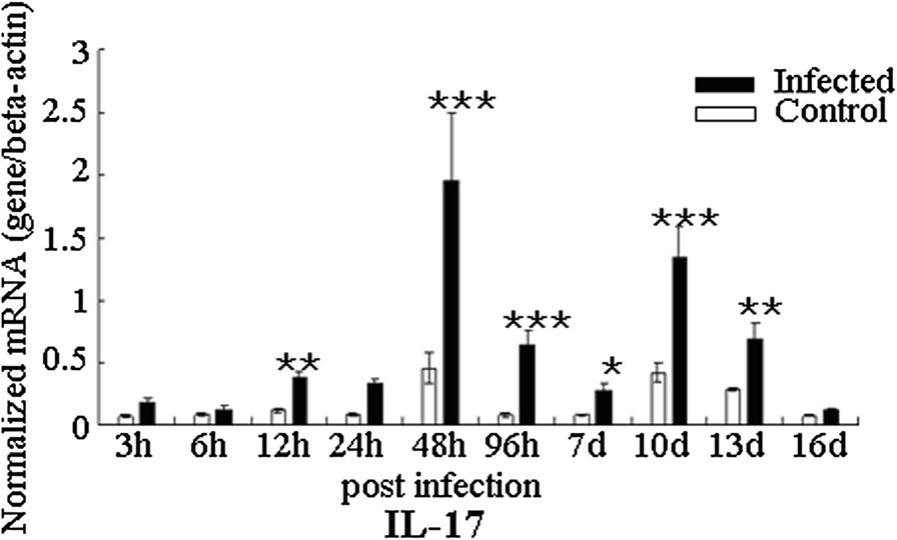
**Expression of IL-17 in spleen in *C. baileyi *infected chickens.** Chickens were orally infected with 1 × 10^5^ oocysts of *C. baileyi*. Spleens were isolated from infected and non-infected chickens at the indicated times post-infection and mRNA levels were determined by qPCR (normalized to beta-actin transcript levels). Each bar represents the mean ± SD values (n = 5). ^*^means *P* < 0.05, ^**^means *P* < 0.01, ^***^means *P* < 0.001.

In addition, we also examined expression of the IL-17 and Th17 response relative cytokines in the transcription level in spleen during the infection (Figures 3 and 4). The expression levels of IL-1β and IL-6 were up-regulated at 12 h pi and 24 h pi during the early phase of the infection, and then they had a peak at 10 d pi and 96 h pi, respectively (Figure 3). The up-regulation of IL-17 mRNA level in infected chickens was observed at 48 h pi, 96 h pi, 10 d pi and 13 d pi compared with that in uninfected controls (Figure 4). For the TGF-β, the mRNA level was robustly up-regulated at 24 h pi and moderately up-regulated 96 h pi and 7 d pi. However, unique to all the molecules examined in spleen, STAT3 transcription level was only significantly increased at 96 h pi during the infection.

Although IL-17 is known to participate in the induction of inflammation during infection of some intracellular protozoan parasites, such as *Toxoplasma gondii *[29], *Leishmania amazonensis *[41], *Trypanosoma cruzi *[27,42] and *Eimeria* spp. [32,43,44], the pathogenic or protective roles of IL-17 in infection by other intracellular protozoan parasites are not well understood. In this study, we studied expression dynamics of IL-17 and Th17 relative cytokines at various time-points after *C. baileyi* infection. The mRNA level of IL-17 in trachea was up-regulated from 12 h pi, with peaks at 12 h, 24 h, and 10 d pi, while the mRNA level of IL-17 in spleen was with peaks at 48 h pi and 10 d pi. These results suggested that the IL-17 may participate in the innate and adaptive immune responses against *C. baileyi* infection.

Biological functions of IL-17 and its roles in disease have been intensively reviewed [45]. IL-17 has an important role in maintaining the mucosal barrier integrity, and it enhances the synthesis of the tight junction protein claudin to strengthen the connections between epithelial cells [46]. Zhang *et al*. [32] reported that IL-17 might induce immunopathology during *Eimeria tenella* infection in chickens. In their study, IL-17 has a robust up-regulation at 6 h pi during the early infection, but the neutralized IL-17 resulted in significantly enhanced weight gains, reduced fecal oocyst shedding, and reduced cecal lesion scores compared with control [32]. However, there were also some studies suggesting that Th17 response played a central role in regulating parasite-induced diseases [42,47,48]. *C. baileyi* is able to establish itself in the mucosal epithelium of a wide variety of organs [35], with the trachea as the most common parasitic site to present inflammation and clinical signs. In the present study, mild clinical symptoms were firstly observed at 8 d pi in the infected group, and affected individuals presented dyspnoea, depression and loose stools. At 10 d pi, all chickens were seen with respiratory symptoms. Dead chickens were also found in the infected group at day 10 pi and 12 pi. Autopsy of dead chickens revealed inflammation and excessive mucus in tracheas. qPCR analysis showed that expression of IL-17 and Th17 response relative cytokines (IL-1β, IL-6 and TGF-β) were up-regulated in tracheas during the course of infection, with the highest levels at day 10 pi. These results suggested that IL-17 may be one of the causes in inducing pathogenic lesions in *Cryptosporidium* infection.

## Conclusions

The present study examined the expression dynamics of IL-17 and Th17 response relative to cytokines after infection with *C. baileyi*. During oral experimental *C. baileyi* infection, IL-17 and related cytokine expression were up-regulated in trachea and spleen of chickens after infection, with the highest levels in tracheas at day 10 pi when the peak oocyst shedding and pathogenic lesions were observed, which suggested that IL-17 may play a role in immunity against *Cryptosporidium* infection. These results would provide basic information for studying pathogenic mechanisms of *Cryptosporidium* spp. and immunity of host against *Cryptosporidium* infections. However, due to few commercial monoclonal antibodies being available for IL-17 and Th17 responses relative cytokines in chickens, immunological methods, such as ELISA and immunohistochemical, are difficult to develop and use. Therefore, to further confirm the role of Th17 cells in immunity in cryptosporidiosis, we will develop monoclonal antibodies for IL-17 of chicken, and study the expression of these cytokines in protein level and the number of Th17 cells during infection in the future.

## Competing interests

The authors declare that they have no competing interests.

## Authors’ contributions

GHZ and SKY conceived and designed the study, and critically revised the manuscript. WYC, WW, YQJ, SZD and YQF performed the experiments, analyzed the data and drafted the manuscript. All authors read and approved the final manuscript.

## Supplementary Material

Additional file 1 **The oocyst shedding patterns of chickens after inoculation with 1 × 10**^**5 **^***C. baileyi *oocysts.** Click here for file
