# Supplementary figures and images for: The expression dynamics of IL-17 and Th17 response relative cytokines in the trachea and spleen of chickens after infection with Cryptosporidium baileyi
Source: Parasit Vectors. 2014 May 6;7:212. doi: 10.1186/1756-3305-7-212 (PMC4036416; doi:10.1186/1756-3305-7-212)

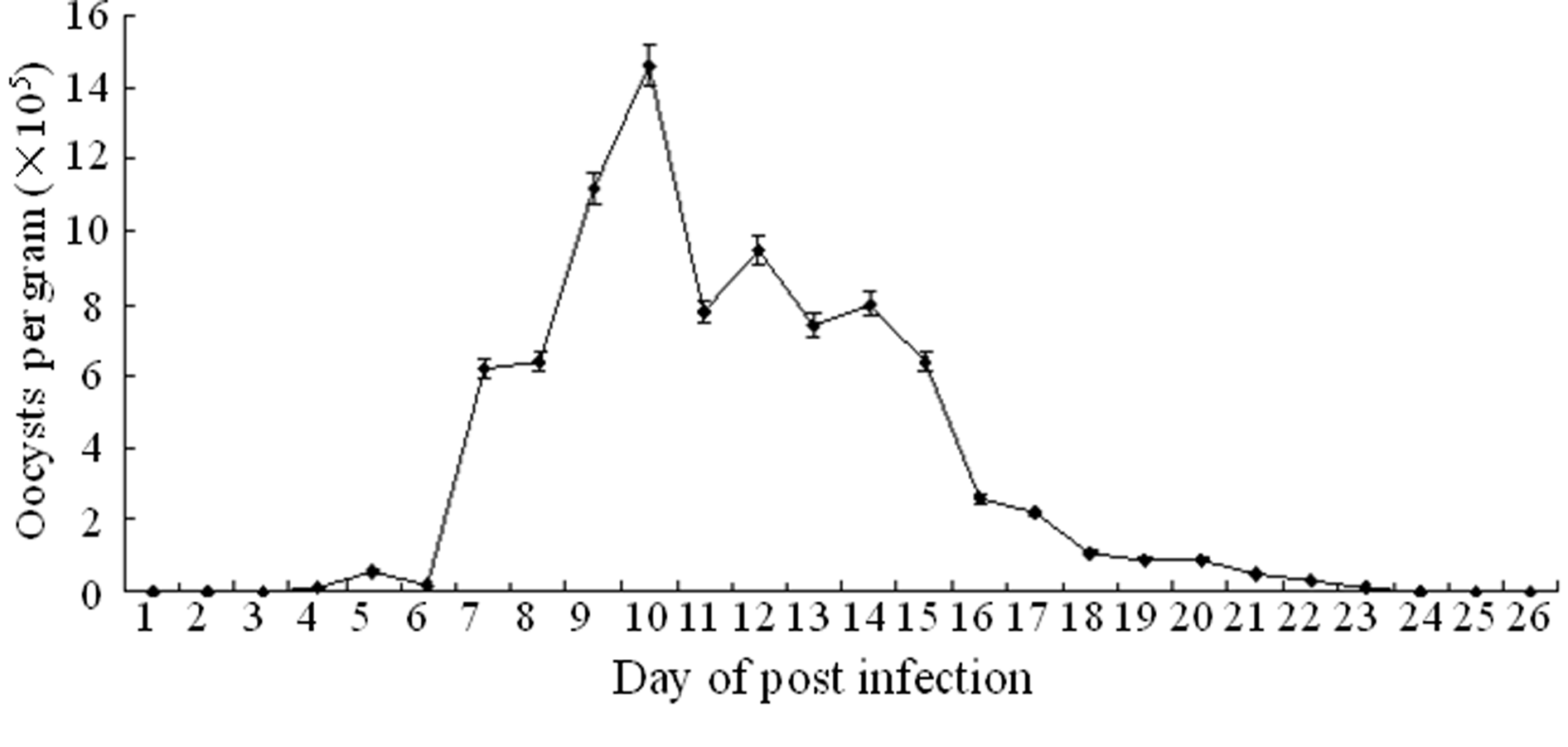

Supplement: Additional file 1 — The oocyst shedding patterns of chickens after inoculation with 1 × 10^5^ C. baileyi oocysts. [file 1756-3305-7-212-S1.tif]
